# Event-Driven Random Back-Propagation: Enabling Neuromorphic Deep Learning Machines

**DOI:** 10.3389/fnins.2017.00324

**Published:** 2017-06-21

**Authors:** Emre O. Neftci, Charles Augustine, Somnath Paul, Georgios Detorakis

**Affiliations:** ^1^Neuromorphic Machine Intelligence Laboratory, Department of Cognitive Sciences, University of California, IrvineIrvine, CA, United States; ^2^Circuit Research Lab, Intel CorporationHilsboro, OR, United States

**Keywords:** spiking neural networks, backpropagation algorithm, feedback alignment, embedded cognition, stochastic processes

## Abstract

An ongoing challenge in neuromorphic computing is to devise general and computationally efficient models of inference and learning which are compatible with the spatial and temporal constraints of the brain. One increasingly popular and successful approach is to take inspiration from inference and learning algorithms used in deep neural networks. However, the workhorse of deep learning, the gradient descent Gradient Back Propagation (BP) rule, often relies on the immediate availability of network-wide information stored with high-precision memory during learning, and precise operations that are difficult to realize in neuromorphic hardware. Remarkably, recent work showed that exact backpropagated gradients are not essential for learning deep representations. Building on these results, we demonstrate an event-driven random BP (eRBP) rule that uses an error-modulated synaptic plasticity for learning deep representations. Using a two-compartment Leaky Integrate & Fire (I&F) neuron, the rule requires only one addition and two comparisons for each synaptic weight, making it very suitable for implementation in digital or mixed-signal neuromorphic hardware. Our results show that using eRBP, deep representations are rapidly learned, achieving classification accuracies on permutation invariant datasets comparable to those obtained in artificial neural network simulations on GPUs, while being robust to neural and synaptic state quantizations during learning.

## 1. Introduction

Biological neurons and synapses can provide the blueprint for inference and learning machines that are potentially 1,000-fold more energy efficient than mainstream computers. However, the breadth of application and scale of present-day neuromorphic hardware remains limited, mainly by a lack of general and efficient inference and learning algorithms compliant with the spatial and temporal constraints of the brain.

Thanks to their general-purpose, modular, and fault-tolerant nature, deep neural networks and machine learning has become a popular and effective means for executing a broad set of practical vision, audition and control tasks in neuromorphic hardware (Esser et al., 2016; Lee et al., 2016; Neftci E. et al., 2016). One outstanding question is whether the learning phase in deep neural networks can be efficiently carried out in neuromorphic hardware as well. Performing such learning on-the-fly is appealing in less controlled environments where no prior, representative dataset exists, and can confer more fine-grained context awareness to behaving cognitive agents. However, the workhorse of deep learning, the gradient descent BP rule, commonly relies on the immediate availability of network-wide information stored with high-precision memory. In digital computers, the access to this information funnels through the von Neumann bottleneck, which dictates the fundamental limits of the computing substrate. Distributing computations along multiple cores in GPUs is an effective solution to mitigate this problem, but even there the scalability of gradient backpropagation in neural networks can sometimes be limited by its data and memory-intensive operations (Seide et al., 2014; Zhu et al., 2016), and more so in the case of fully connected networks (Seide et al., 2014).

The implementation of Gradient Back Propagation (hereafter BP for short) on a neural substrate is even more challenging (Grossberg, 1987; Baldi et al., 2016; Lee et al., 2016) because it requires (1) using synaptic weights that are identical with forward passes (symmetric weights requirements, also known as the weight transport problem), (2) carrying out the operations involved in BP including multiplications with derivatives and activation functions, (3) propagating error signals with high, floating-point precision, (4) alternating between forward and backward passes, (5) changing the sign of synaptic weights, and (6) availability of targets (labels). While some recent work in neural networks shows that error signals can be propagated using low precision (sometimes down to 1 bit; Courbariaux and Bengio, 2016; Rastegari et al., 2016), the essence of these challenges is that BP often requires precise linear and non-linear transformations and information that is not local to the computational building blocks in a neural substrate, meaning that special communication channels must be provisioned (Baldi and Sadowski, 2015). Whether a given operation is local or not depends on the physical implementation that carries out the computations. For example, while symmetric weights in neural networks are compatible with von Neumann architectures (and even desirable since weights in both directions are shared), the same is not true in a distributed system such as the brain: elementary computing units do not have bidirectional connections with the same weight in each direction. Since neuromorphic implementations generally assume dynamics closely related to the those in the brain, requirements (1–4) above also hinder efficient implementations of BP in neuromorphic hardware.

Although, previous work (Lee et al., 2016; Lillicrap et al., 2016; O'Connor and Welling, 2016) overcomes some of the fundamental difficulties of gradient BP listed above in spiking networks, here we tackle all of the key difficulties using event-driven random BP (eRBP), a synaptic plasticity rule for deep spiking neural networks achieving classification accuracies that are similar to those obtained in artificial neural networks, potentially running on a fraction of the energy budget with dedicated neuromorphic hardware.

eRBP builds on the recent advances in approximate forms of the gradient BP rule (Lee et al., 2014; Liao et al., 2015; Baldi et al., 2016; Lillicrap et al., 2016) for training spiking neurons of the type used in neuromorphic hardware to perform supervised learning. These approximations solve the non-locality problem by replacing weights in the backpropagation phase with random ones, leading to remarkably little loss in classification performance on benchmark tasks (Baldi et al., 2016; Lillicrap et al., 2016) (requirement 1 above). Although, a general theoretical understanding of random BP (RBP) is still a subject of intense research, extended simulations and analyses of linear networks show that, during learning, the network adjusts its feed-forward weights such that they align with the (random) feedback weights, which is arguably equally good in communicating gradients. eRBP is an asynchronous (event-driven) adaptation of random BP that can be tightly embedded with the dynamics of dual compartment I&F neurons that costs one addition and two comparisons per synaptic weight update. Extended experimentations show that the spiking nature of neuromorphic hardware and the lack of general linear and non-linear computations at the neuron does not prevent accurate learning on classification tasks (requirement 2, 3), and operates continuously and asynchronously without alternation of forward or backward passes (requirement 4). Additional experimental evidence shows that eRBP is robust to fixed width representations of the synaptic weights, making it suitable for dedicated neuromorphic hardware.

The focus of eRBP is to achieve real-time, online learning at higher power efficiency compared to deep learning on standard hardware, rather than achieving the highest accuracy on a given task. The success of eRBP on these measures lays out the foundations of neuromorphic deep learning machines, and paves the way for learning with streaming spike-event data in neuromorphic platforms at proficiencies close to those of artificial neural networks.

This article is organized as follows: key theoretical and simulation results are provided in the results sections, followed by a general discussion and conclusion. Technical details of eRBP and its implementation are provided as the final section.

## 2. Results

### 2.1. Event-driven random backpropagation

The central contribution of this article is event-driven RBP (eRBP), a presynaptic spike-driven plasticity rule modulated by top-down errors and gated by the state of the postsynaptic neuron. The idea behind this additional modulation factor is motivated by supervised gradient descent learning in artificial neural networks and biologically plausible models of three-factor plasticity rules (Urbanczik and Senn, 2014), which were argued to subserve supervised, unsupervised and reinforcement learning, an idea that was also reported in Lillicrap et al. (2016).

In gradient descent using a squared error cost function, weight updates for a neuron in layer *l* are computed as:

(1) $$\Delta w_{ij}(t)=y_{j}(t)\phi^{\prime }\left(\sum_{j}w_{ij}(t)y_{j}(t)\right)T_{i}(t)$$

where *y*_*j*_ is the presynaptic activity and ϕ is the activation function of the neuron. In standard BP, the term *T*_*i*_ is computed using the backpropagated errors (see Section 5), while in the RBP rule used here, it is computed using a direct random linear combination of the errors,

$$T_{i}(t)=\sum_{k}e_{k}(t)g_{ik}$$

where *e*_*k*_ is the error of output neuron *k*, and *g*_*ik*_ are the fixed random feedback weights to the hidden layer neuron *i*. In the methods, we show that eRBP is a spatially and temporally local rule that implements random backpropagation in an event-driven fashion. The eRBP dynamics for synapse *j* of neuron *i* can be summarized as follows:

(2) $$\Delta w_{ij}(t)=T_{i}(t)\Theta (I_{i}(t))S_{j}^{pre}(t)$$

where $S_{j}^{pre}\left(t\right)$ represents the spike train of presynaptic neuron *j*, and Θ is the derivative of the spiking neuron's activation function evaluated at the total synaptic input *I*_*i*_. While the weight updates depend on the error at the output, which is non-local, this information needs only to be transmitted on a neuron-to-neuron basis (rather than each synapse, which would be prohibitive). This error gradient signal is then maintained at an auxiliary state of the neuron, whose average value encodes *T*_*i*_, thus making the error gradient available to its synapses during learning. For the final output (prediction) layer, *T*_*i*_ is proportional to the classification error (*e*_*i*_) of the considered neuron, similarly to the standard delta rule. For hidden layers, the *T*_*i*_ is proportional to the error projected randomly to the hidden neurons, i.e., to $T_{i}≅\underset{k}{\sum }e_{k}g_{ik}$ as in Equation (1). We found that a boxcar function in place of Θ provides very good results, while being more amenable to hardware implementation compared to the alternative of computing the exact derivative of the activation function.

$$\Theta (I_{i})≅\{\begin{array}{l} 1\text{ if }b_{min}<I_{i}<b_{max}\\ 0\text{ otherwise} \end{array}.$$

This choice is motivated by the fact that the activation function of I&F neurons with absolute refractory period can be approximated by a linear threshold unit (also known as rectified linear unit) with saturation whose derivative is exactly the boxcar function. In this case, the eRBP synaptic weight update consists of additions and comparisons only, and can be captured using the following operations for neuron *i*:

**    function** eRBP

**         for***k* ∈ {presynaptic spike indices **S**^*pre*^}**do**

              **if** *b*_*min*_ < *I*_*i*_ < *b*_*max*_ **then** *w*_*ik*_ ← *w*_*ik*_ + *T*_*i*_,

**              end if**

**        end for**

**   end function**

where **S**^*pre*^ is the list of presynaptic neuron indices that have spiked, *T*_*i*_ is the linear combination of the error vector. In the spiking network, *T*_*i*_ is proportional to the state of an auxiliary compartment that integrates spikes from the error neurons. The above pseudocode states that, in eRBP, a weight update is performed only when a presynaptic neuron fires. The eRBP rule is demonstrated in two different stochastic network configurations: one where noise is additive, another where noise is multiplicative, where all plastic synapses can fail to generate a post-synaptic potential with a fixed probability (blank-out probability, see ξ(*t*) in Equation 18).

Provided the second compartment dynamics, no multiplications are necessary for an eRBP update. This second compartment can be disabled after learning without affecting the inference dynamics. This rule is reminiscent of membrane voltage-based rules, where spike-driven plasticity is induced only when membrane voltage is inside an eligibility window (Brader et al., 2007; Chicca et al., 2013).

The realization of eRBP on neuromorphic hardware requires an auxiliary learning variable for integrating and storing top-down error signals during learning, which can be substantiated by a dendritic compartment. Provided this variable, each synaptic weight update incurs only two comparison operations and one addition. Additions and comparisons can be implemented very naturally in neuromorphic VLSI circuits (Liu et al., 2002), and costs in the order of tens of femtojoules in digital circuits (45 *nm* processes; Horowitz, 2014). In practice, synapses outnumber neurons by a factor of 100 or more, hence the cost of a second compartment dynamics will be small in general compared to the cost of the synaptic update. As a concrete example we use leaky, two compartment, current-based Integrate-and-Fire neurons with additive and multiplicative noise and linear synapses (see Section 5). The gating term Θ, implemented as two comparisons, operates on the total synaptic input. This choice is guided by the gradient descent rule, which dictates that the derivative should be evaluated on the total input (Section 5). The linearity of the synaptic dynamics allows to use a single dynamical variable for all synapses, such that the value of this dynamical variable is exactly equal to the total synaptic input *I*_*i*_, and thus readily available at the neuron and the synapses.

### 2.2. Spiking networks equipped with eRBP learn with high accuracy

**Figure 1:**
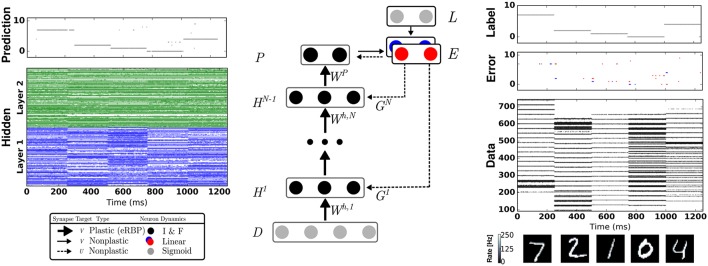
Network Architecture for Event-driven Random Backpropagation (eRBP) and example spiking activity after training a 784-200-200-10 network for 60 epochs. The network consists of feed-forward layers (*H*^1^, …, *H*^*N*^) for prediction and feedback layers for supervised training with labels (targets) *L*. Full arrows indicate synaptic connections, thick full arrows indicate plastic synapses, and dashed arrows indicate synaptic plasticity modulation. In this example, digits 7, 2, 1, 0, 4 were presented in sequence to the network. The digit pixel values are transformed to spike trains (layer D) using a Spike Response Model (Equation 21). Neurons in the network indicated by black circles were implemented as two-compartment I&F neurons (Equations 18 and 20). The error is the difference between labels (L) and predictions (P), and is implemented using a pair of neurons coding for positive error (blue) and negative error (red), following Equation (17). Each hidden neuron receives inputs from a random combination of the pair of error neurons to implement random BP. Output neurons receive inputs from the pair of error neurons in a one-to-one fashion. At the moment of data sample (digit) transitions, bursts of activity (about 3 spikes) in the error neurons occur. To prevent the perturbation of the weights during these transitions, no weight updates were undertaken immediately after changing data sample.

**Figure 2:**
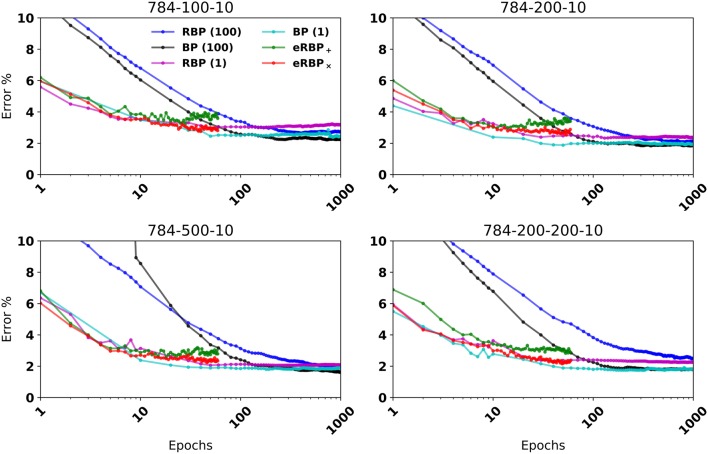
MNIST Classification error on fully connected artificial neural networks (BP and RBP) and on spiking neural networks (eRBP). Curves for eRBP were obtained by averaging across 5 simulations with different seeds.

We demonstrate eRBP in networks consisting of one and two hidden layers trained on permutation invariant MNIST and EMNIST (Table 1, Figures 1, 2), although eRBP can in theory generalize to other datasets, tasks and network architectures as well. Rather than optimizing for absolute classification performance, we compare to equivalent artificial (non-spiking) neural networks trained with RBP and standard BP, with free parameters fine-tuned to achieve the highest accuracy on the considered classification tasks (Table 1).

**Table 1:** Classification error on the permutation invariant MNIST task (test set) obtained by averaging test errors of the last 5 epochs (for MNIST) and last epoch for EMNIST.

**Network**	**Classification error**					
**Dataset**	**eRBP_+_ (%)**	**eRBP_×_ (%)**	**RBP (100) (%)**	**RBP (1) (%)**	**BP (100) (%)**	**BP (1) (%)**
PI MNIST 784-100-10	3.77 (3.23)	2.89 (2.81)	2.74 (2.64)	3.19 (2.98)	2.25 (2.19)	2.44 (2.39)
PI MNIST 784-200-10	3.53 (2.98)	2.78 (2.53)	2.13 (2.04)	2.37 (2.33)	1.85 (1.78)	1.94 (1.88)
PI MNIST 784-500-10	2.86 (2.57)	2.34 (2.23)	2.00 (1.96)	2.09 (2.06)	1.63 (1.60)	1.88 (1.80)
PI MNIST 784-200-200-10	2.96 (2.85)	2.29 (2.22)	2.50 (2.45)	2.26 (2.25)	1.80 (1.78)	1.82 (1.74)
PI MNIST 784-500-500-10	2.36 (2.28)	2.02 (1.96)	2.24 (2.0)	2.34 (2.31)	1.90 (1.86)	1.69 (1.56)
PI EMNIST 784-200-200-10	26.76 (25.26)	21.83 (21.4)	22.3 (20.18)	32.37 (26.48)	18.42 (16.06)	18.23 (17.72)

*Numbers in parentheses indicates the value of n_batch_. Peak accuracies were reported for GPU based simulations. Value between parentheses indicate the highest measured test accuracies*.

When equipped with stochastic connections (multiplicative noise) that randomly blank out presynaptic spikes, the network performed better overall (labeled *eRBP*_×_). In 2-hidden layer network configurations eRBP_×_ achieved performances on par with those achieved with RBP in artificial networks. Test error with additive noise (eRBP_+_) had a tendency to increase after epoch 15, which is likely due to overfitting. This did not occur with eRBP_×_ and is consistent with the regularizing effect of stochasticity in neural networks (Hinton et al., 2012; Baldi and Sadowski, 2013; Wan et al., 2013). We trained the spiking neural network on a more difficult task consisting of digits and letters (EMNIST) (Cohen et al., 2017), collectively accounting up to 47 classes. The spiking networks consisting of 400 hidden neurons equipped with multiplicative noise and eRBP achieved errors similar to those obtained using the online ELM-based classifier consisting of 10,000 hidden neurons used in (Cohen et al., 2017). The relative gaps is accuracy between eRBP and BP are in the same range as in the case with MNIST.

The reasons why the eRBP_×_ performs better than the eRBP_+_ configuration cannot only be attributed to its regularizing effect: As learning progresses, a significant portion of the neurons tend to fire near their maximum rate and synchronize their spiking activity across layers as a result of large synaptic weights (and thus presynaptic inputs). Synchronized spike activity is not well captured by firing rate models, which is assumed by eRBP (see Section 5). Additive noise has a relatively small effect when the magnitude of the presynaptic input is large. However, multiplicative blank-out noise improves learning by introducing irregularity in the presynaptic spike-trains even when presynaptic neurons fire regularly. This type of “always-on” stochasticity also was argued to approximate Bayesian inference with Gaussian processes (Gal and Ghahramani, 2015). In summary, the eRBP_×_ yields higher accuracy at a small overhead in random variable generation, and is thus the configuration of choice compared to eRBP_+_. Results for eRBP_+_ are provided for comparison or as a reference for existing neuromorphic hardware that do not support multiplicative noise. For comparison purposes, we show training with *n*_*batch*_ = 1, whose end performance is often slightly better and learning nearly as quickly as eRBP. Since it is very inefficient to train with *n*_*batch*_ = 1 on GPUs and practically impossible to scale to larger problems, we will base discussion on the standard *n*_*batch*_ = 100 unless otherwise stated.

Overall, the learned classification accuracy with eRBP_×_ is close to that obtained with offline training of neural networks (e.g., GPUs, *n*_*batch*_ = 100) using RBP. Thus, the gap between eRBP and BP can be largely attributed to the approximate RBP gradient. On the other hand, eRBP is a simple, local synaptic plasticity rule that is entirely event-based. This is in contrast to recent work in training spiking neural networks using standard gradient backpropagation (Lee et al., 2016), where errors are transmitted as real values across the backpropagated chain path. At least two advantages accrue from eRBP: (1) its implementation can be largely achieved using *existing* asynchronous neuromorphic technologies (Vogelstein et al., 2002; Chicca et al., 2013; Park et al., 2014), (2) no error is transmitted and no weights are updated if the error is below the firing threshold of the error neurons. The event-based implementation of eRBP thus enables an end-to-end asynchronous, event-based implementation of deep learning in neuromorphic hardware.

#### 2.2.1. eRBP learning dynamics

**Figure 3:**
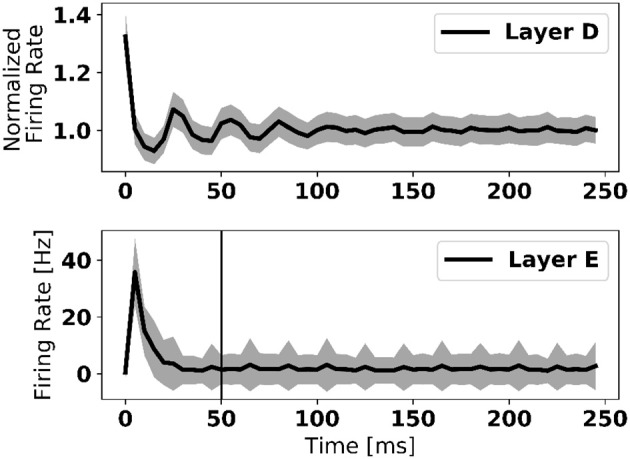
Firing rate of data layer and error layer upon stimulus onset, averaged across 1,000 trials and all neurons in the layer. The large firing rate at the onset is caused by synchronized neural activity. The vertical line in the bottom figure depicts the 50 *ms* after which learning is enabled after each digit presentation. This duration is selected as a conservative measure to present transitory dynamics in the network to corrupt the error feedback. Shaded area is one standard deviation across all 1,000 trials.

Transitions between two data samples of different class (digit) are marked by bursts of activity in the error neurons (Figure 1). To overcome this problem, weight updates were disabled the first 50 *ms* after the new digit onset. Figure 3 shows that about 20 ms are necessary for the error neurons to reach their stable activity after the onset of the input in a three-layer network—a duration that we refer to as “loop duration.” The vertical line in Figure 3 marks the chosen 50 *ms*, which is a conservative estimate of the loop duration. The speed of eRBP learning is thus limited by the loop duration, which depends on the time constants of the synapses and neurons, as well as the number of layers in the network. This implies that in deep networks, the weight updates must be disabled for durations that scale with the number of layers. We note that this problem, also referred to as “update locking” is also faced in standard implementations, which can be solved by pipelining the forward and backward passes (Gadea et al., 2000) or by estimating error gradients before they are computed using the output layer (Jaderberg et al., 2016; Czarnecki et al., 2017). The latter method is compatible with eRBP in principle as the authors demonstrated it using feedback alignment, and can provide a natural solution to this problem.

In future work involving practical applications on autonomous systems, it will be beneficial to interleave learning and inference stages without explicitly controlling the learning rate. One way to achieve this is to introduce a negative bias in the error neurons by means of a constant negative input and an equal positive bias in the label neurons such that the error neuron can be only be active when an input label is provided^1^. The same solution can overcome the perturbations caused by bursts of error activity during digit transitions (see red and blue spikes in Figure 1).

The presence of these bursts of error activity suggest that eRBP could learn spatiotemporal sequences as well. However, learning useful latent representations of the sequences requires solving a temporal credit assignment problem at the hidden layer—a problem that is commonly solved with gradient BP-through-time in artificial neural networks (Rumelhart et al., 1988)—which could be tackled using synaptic eligibility dynamics based on ideas of reinforcement learning (Sutton and Barto, 1998) or synthetic gradients (Jaderberg et al., 2016; Czarnecki et al., 2017).

### 2.3. Classification with single spikes is highly accurate and efficient

**Figure 4:**
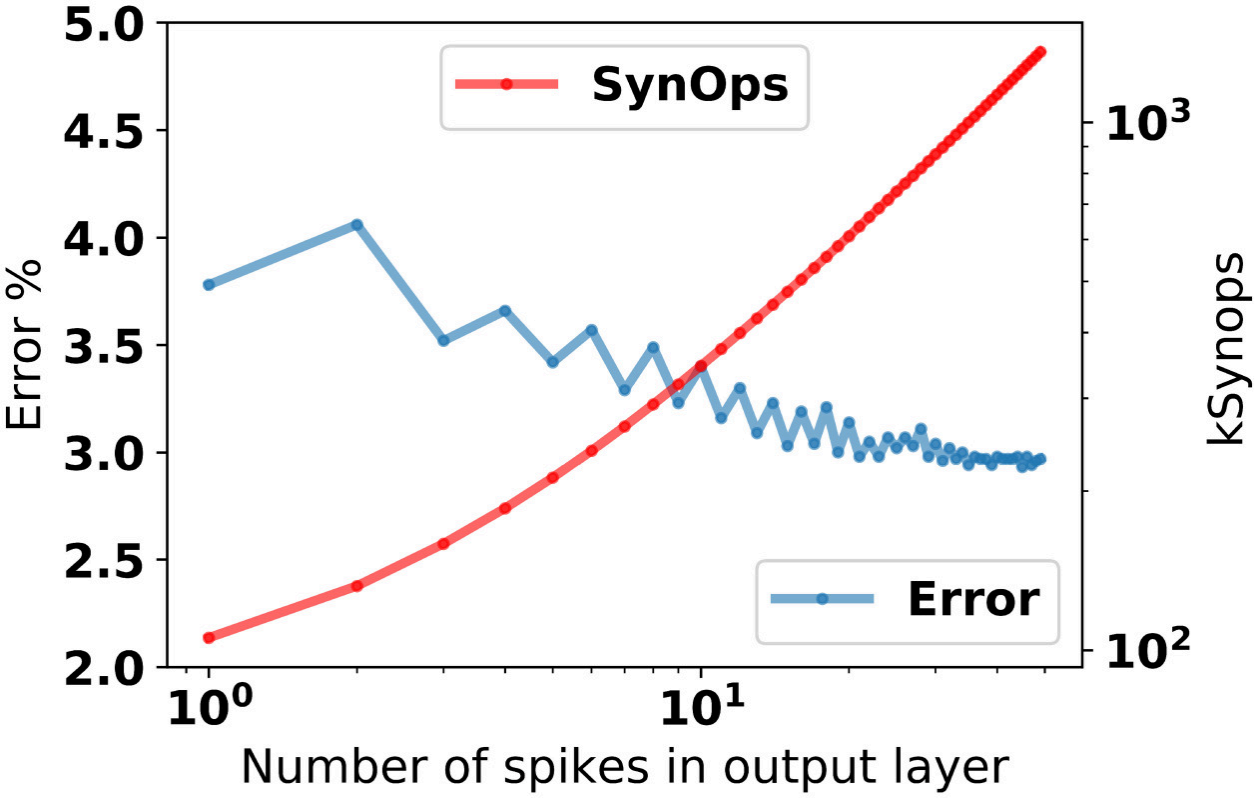
Classification error in the 784-200-10 eRBP_+_ network as a function of the number of spikes in the prediction layer, and total number of synaptic operations incurred up to each output spike. To obtain this data, the network was first stimulated with random patterns, and the spikes in the output layer were counted after τ_*syn*_ = 4 *ms*.

The response of the 784-200-10 network after stimulus onset is about one synaptic time constant. Using the first spike after 2τ_*s*_ = 8*ms* from the stimulus onset for classification leads to about 4% error (Figure 4), and improves steadily as the number output layer spikes increase.

In this example, classification using the first spike incurred about 100 *k* synaptic operations (averaged over 10,000 test samples), most of which occur between the data and the hidden layer (784 neurons and 200 neurons, respectively). In existing dedicated neuromorphic hardware (Merolla et al., 2014; Park et al., 2014; Qiao et al., 2015), the energetic cost of a synaptic operation is about 20*pJ*. On such hardware, single spike classification in eRBP trained networks can potentially result in about 2μ*J* energy per classification. This figure is comparable to the state-of-the-art in digital neuromorphic hardware (≅2 μ*J* at this accuracy; Esser et al., 2015) and potentially 1,000x more efficient than current GPU technology (>*mJ*). We note that no sparsity criterion was enforced in this network. We expect that sparsity implemented explicitly using weight regularization or implicitly using Dropout or DropConnect techniques (Baldi and Sadowski, 2013) can further reduce this energy, by virtue of the lower activity in the hidden layer.

The low latency response with high accuracy may seem at odds with the inherent firing rate code underlying the network computations (see Section 5). However, a code based on the time of the first-spike is consistent with a firing rate code, since a neuron with a high firing rate is expected to fire first (Gerstner and Kistler, 2002). In addition, the onset of the stimulus provokes a burst of synchronized activity, which further favors the rapid onset of the prediction response. These results suggest that despite the underlying firing rate code, eRBP can take advantage of the spiking dynamics, with classification accuracies comparable to spiking networks trained exclusively for single-spike classification (Mostafa, 2016).

### 2.4. Spiking networks equipped with eRBP learn rapidly and efficiently

In the spiking simulations, weight updates are updated during the presentation of *each* digit. This is in strong contrast to batch gradient descent, where weight updates are computed across the entire dataset, or more commonly, across random minibatches of the dataset. We observe that the spiking neural network requires fewer iterations of the dataset to reach the peak classification performance compared to the artificial neural network trained with batch gradient descent (Figure 2, *n*_*batch*_ = 100). Batch or minibatch learning improves learning speed in conventional hardware thanks to vectorization libraries or efficient parallelization with GPUs' SIMD architecture, and leads to smoother convergence. However, this approach results in *n*_*batch*_ times fewer weight updates per epoch compared to online gradient descent. In contrast, the spiking neural network is updated multiple times during each sample presentation, and accounts in large part for the faster convergence of learning: Running GPU-based simulations using *n*_*batch*_ = 1 resulted in a similar speed of convergence with the spiking neural networks, and in some cases improved the final accuracy, most likely due to the added stochasticity. Other spiking networks trained online using stochastic gradient descent, i.e., updates after each image presentation, achieved comparable speedup (Lee et al., 2016; O'Connor and Welling, 2016).

These results are not entirely surprising since seminal work in stochastic gradient descent established that with suitable conditions on the learning rate, the solution to a learning problem obtained with stochastic gradient descent is asymptotically as good as the solution obtained with batch gradient descent (Le Cun and Bottou, 2004) for a given number of samples. Furthermore, for equal computational resources, online gradient descent can process more data samples (Le Cun and Bottou, 2004), while requiring less memory for implementation. Thus, for an equal number of compute operations per unit time, online gradient descent converges faster than batch learning. Learning with *n*_*batch*_ = 1 in GPUs is much slower because fewer operations are vectorized across data samples. We observe more than 50x performance hit by switch to *n*_*batch*_ = 1 in the GPU-based experiments presented in Table 1.

It is fortunate that synaptic plasticity is inherently “online” in the machine learning sense, given that potential applications of neuromorphic hardware often involve real-time streaming data.

#### 2.4.1. Efficiency in learning: achieving SynOp-MAC parity

**Figure 5:**
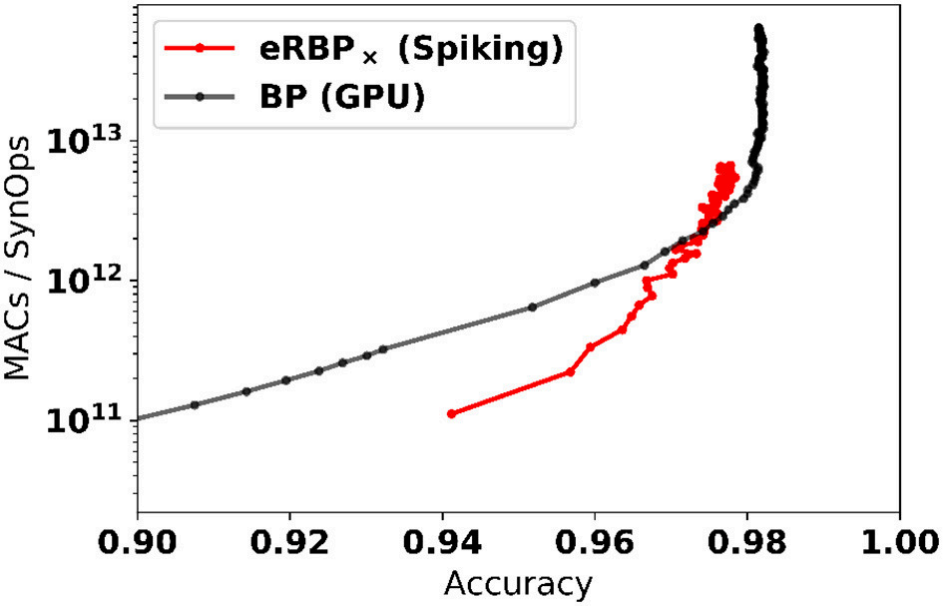
Spiking Neural Networks equipped with eRBP with stochastic synapses (multiplicative noise) achieve SynOp-MAC parity at the MNIST task. The number of multiply-accumulate (MAC) operations required for reaching a given accuracy is compared to the number of synaptic operations (SynOps) in the spiking network for the MNIST learning task (784-200-200-10 network). Both networks requires roughly the same number of operations to reach the same accuracy during learning. Only MACs incurred in the matrix multiplications are taken into account (other necessary operations e.g., additions, logistic function calls, and weight updates were not taken into account here, and would further favor the spiking network).

The online, event-based learning in eRBP combined with the reduced number of required dataset iterations suggests that learning on neuromorphic hardware can be particularly efficient. Furthermore, in neuromorphic hardware, only active connections in the network incur a SynOp. To demonstrate the efficiency of the learning, we report the number of multiply-accumulate (MAC) operations required for reaching a given accuracy compared to the number of synaptic operations (SynOps) in the spiking network for the MNIST learning task (784-200-200-10 network, Figure 5). For computing the number of MACs in the GPU based simulation, we used minibatch learning since online learning (sample by sample) is highly inefficient on GPUs. We find that both networks require roughly the same number of operations to reach the same accuracy during learning. This SynOp–MAC parity was also reported in synaptic sampling machines (Neftci E. O. et al., 2016). There, it was argued that SynOp-MAC parity is very promising for hardware implementations because a SynOp in dedicated hardware potentially consumes much less power than a MAC in a general purpose digital processor.

The spiking neural networks learn quickly initially (epoch 1 at 94%), but subsequent improvements become slower compared to the artificial neural network. The reasons for this slowdown are likely due to (1) random backpropagation/direct feedback alignment, (2) spikes emanating from error-coding neurons becoming very sparse toward the end of the training, which prevent fine adjustments of the weight. We speculate that a scheduled or network accuracy-based adjustment of the error neuron sensitivity is likely to mitigate the latter cause. Such modifications, along with more sophisticated learning rules involving momentum and learning rate decay are left for future work.

### 2.5. eRBP can learn with low precision, fixed point representations

The effectiveness of stochastic gradient descent degrades when the precision of the synaptic weights using a fixed point representation is smaller than 16 bits (Courbariaux et al., 2014). This is because quantization determines the smallest learning rate and bounds the range of the synaptic weights, thereby preventing averaging the variability across dataset iterations. The tight integration of memory with computing circuits as pursued in neuromorphic chip design is challenging due to space constraints and memory leakage, thereby constraining the memory that can be attributed to each synaptic weight. For this reason, full precision computer simulations of spiking networks may be unrepresentative of performance that can be attained in dedicated neuromorphic designs due to quantization of neural states, parameters, and synaptic weights.

**Figure 6:**
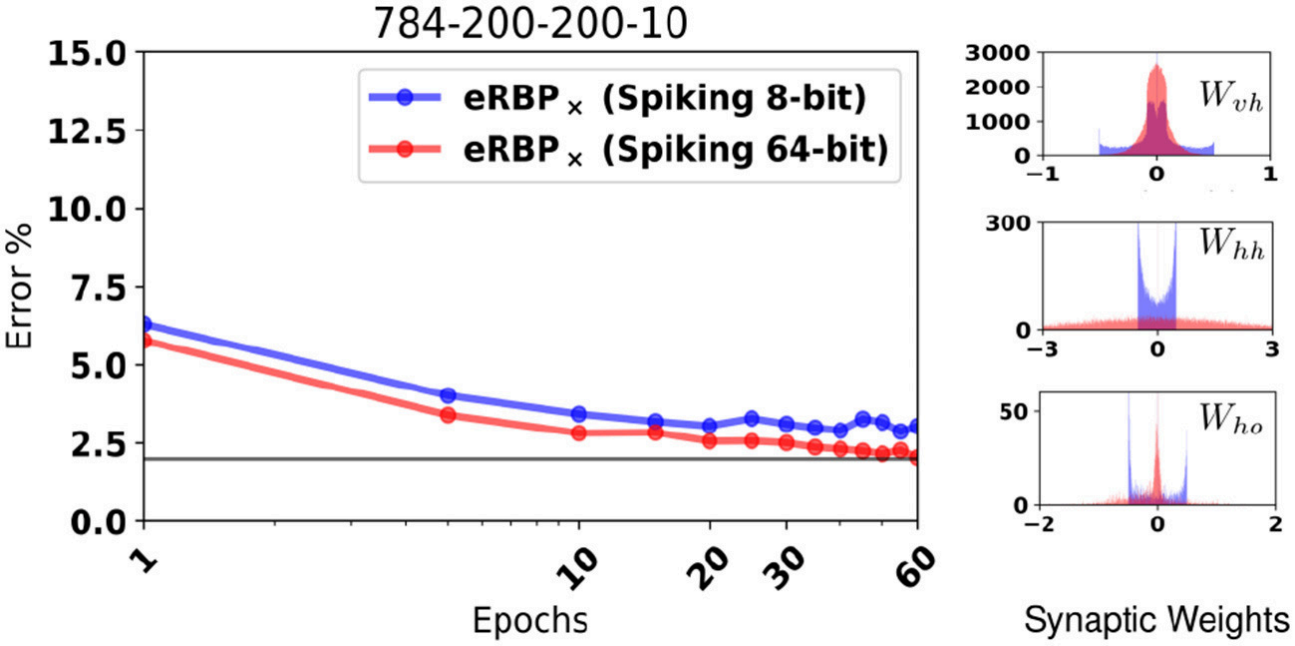
**(Left)** MNIST Classification error using a fully connected 784-200-200-10 network with quantized synaptic weights and rounded using the randomized rounding (Muller and Indiveri, 2015). **(Right)** Histogram of synaptic weights of the quantized network after training.

Extended simulations suggest that the random BP performance at 10 bits precision is indistinguishable from unquantized weights (Baldi et al., 2016), but whether this is the case for online learning has yet been tested. Here, we hypothesize that 8 bit synaptic weight is a good trade-off between the ability to learn with high accuracy and the cost of implementation in future hardware. To demonstrate robustness to such constraints, we test eRBP with randomized rounding (Muller and Indiveri, 2015) and stochastic synapses on the PI MNIST task, where the values that the synaptic weights could take were limited to 256 equally spaced values in [−0.5, 0.5]. Figure 6 shows training with randomized rounding 784-200-200-10 network trained on MNIST compared to no rounding (64 bits). The loss of accuracy using the randomized rounding technique in this configuration is at an acceptable 1%. Histograms show that synaptic weight values tend to converge on a distribution with ranges that depend on the layer. Furthermore, the histogram for the first layer (*W*_*vh*_) shows that randomized rounding tends to further diffuse the weights across the specified range compared to the case without rounding, and increase the tendency for the weights to aggregate at the boundaries. These two effects can in principle be mitigated using weight or batch normalization techniques (Ioffe and Szegedy, 2015; Salimans and Kingma, 2016), where weight distributions were observed to change less during learning. (Lee et al., 2016) have explored such weight normalization through local inhibition mechanisms that effectively change the firing threshold of the neurons, thus the effective synaptic weights.

## 3. Discussion

The gradient descent BP rule is a powerful algorithm that is ubiquitous in deep learning, but when implemented in a neuromorphic substrate, it relies on the immediate availability of network-wide information stored with high-precision memory. More specifically, (Baldi et al., 2016) and (Lee et al., 2016) list several reasons why the following requirements of gradient BP make them biologically implausible. The essence of these difficulties is that gradient BP is non-local in space and in time when implemented on a neural substrate, and requires precise linear and non-linear computations. The feedback alignment work demonstrated that symmetric weights were not necessary for communicating error signals across layers (Lillicrap et al., 2016). Here we demonstrated a learning rule inspired by feedback alignment, and membrane voltage-gated plasticity rules, and three-factor synaptic plasticity rules proposed in the computational neuroscience literature. With an adequate network architecture, we find that the spike-based computations and the lack of general linear and non-linear computations and alternating forward and backward steps does not prevent accurate learning. Although, previous work overcome some of the non-locality problems of gradient BP (Lee et al., 2016; Lillicrap et al., 2016; O'Connor and Welling, 2016), eRBP overcomes all of the key difficulties using a simple rule that incurs one addition and two comparisons per synaptic weight update.

Taken together, our results suggest that general-purpose deep learning using streaming spike-event data in neuromorphic platforms at artificial neural network proficiencies is realizable.

Our experiments target neuromorphic implementations of spiking neural networks with embedded plasticity. Membrane-voltage based learning rules implemented in mixed-signal neuromorphic hardware (Qiao et al., 2015; Huayaney et al., 2016) are compatible with eRBP provided that synaptic weight updates can be modulated by an external signal on a neuron-to-neuron basis. Following this route, and combined with the recent advances in neuromorphic engineering and emerging nanotechnologies, eRBP can become key to ultra low-power processing in space and power constrained platforms.

### 3.1. Why neuromorphic learning machines?

Spiking neural networks, especially those based on the I&F neuron types severely restrict computations during learning and inference. With the wide availability of graphical processing units and future dedicated machine learning accelerators, the neuromorphic spike-based approach to learning machines is often heavily criticized as being misguided. While this may be true for some hardware designs and on metrics based on absolute accuracy at most standardized benchmark tasks, neuromorphic hardware dedicated for embedded learning can have distinctive advantages thanks to: (1) asynchronous, event-based communication, which considerably reduces the communication between distributed processes, (2) natural exploitation of “rate” codes and “spike” codes where single spikes are meaningful, leading to fast and thus power-efficient and gradual responses (Figure 4, see also O'Connor and Welling, 2016), (3) on-line learning, which can naturally support continual (life-long) learning. In addition, the premise of neuromorphic engineering, i.e., that electronic and biological share similar constraints on communication, power, and reliability (Mead, 1990), also extends to the algorithmic domain. That is, accommodating machine learning algorithms within the constraints of ultra-low power hardware for adaptive behavior (i.e., embedded learning) is likely to result in solutions for communication, computations and reliability that are in close resemblance with the brain. The convergence between the two approaches (neuromorphic vs. artificial) will not only improve the design of neuromorphic learning machines, but can also widen the breadth of knowledge transfer between computational neuroscience and deep learning.

Many examples that led to the unprecedented success in machine learning have substantial overlap with equivalent neural mechanisms, such as normalization (Ioffe and Szegedy, 2015; Ren et al., 2016), attention, short-term memory for learning complex tasks (Graves et al., 2014), and memory consolidation through fast replays for reinforcement learning (Mnih et al., 2015; Kumaran et al., 2016). One example relevant to the presented work is the Binarized Neural Network (BNN) (Courbariaux and Bengio, 2016). The BNN is trained such that weights and activities are −1 or 1, which considerably reduces the energetic footprint of inference, because multiplications are not necessary and the memory requirements for inference are much smaller. The discrete, quantized dynamics of I&F neurons shares many similarities with the BNN, such as binary activations (spikes), low-precision variables, and straight-through gradient estimators. Our neurally inspired approach has important and potentially advantageous differences with regard to binarized networks: network activity is sparse and data-driven (asynchronous), random variables for stochasticity are generated only when neurons spike, errors are backpropagated only for misclassified examples, and learning is ongoing leading to accurate, early, single-spike classification.

### 3.2. Relation to prior work in random backpropagation

Our learning rule builds on the feedback alignment learning rule demonstrating that random feedback can deliver useful teaching signals by aligning the feed-forward weights with the feed-back weights (Lillicrap et al., 2016). The authors also demonstrated a spiking neural network implementing feedback alignment, demonstrating that feedback alignment is able to implicitly adapt to random feedback when the forward and backward pathways both operate continuously. However, their learning rule is not event-based as in eRBP, but operates in a continuous-time fashion that is not directly compatible with spike-driven plasticity, and a direct neuromorphic implementation thereof would be inadequate due to the high bandwidth communication required between neurons. Furthermore, their model is a spike response model that does not emulate the physical dynamics of spiking neurons such as I&F neurons. Another difference between eRBP and the network presented in Lillicrap et al. (2016) is that eRBP contains only one error-coding layer, whereas feedback alignment contains one error-coding layer per hidden layer. Such direct feedback alignment was recently proposed in Nø kland (2016) and Baldi et al. (2016), and theoretical analyses demonstrate that gradients computed in this fashion are within 90 degrees of the backpropagated gradient. Baldi et al. (2016) studied feedback alignment in the framework of local learning and the learning channel, and derived several other flavors of random BP such as adaptive, sparse, and indirect RBP, along with their combinations. In related work, Lee et al. (2014) showed how feedback weights can be learned to improve the classification accuracy by training the feedback weights to learn the inverse of the feedforward mapping. After initial submission of this article, Samadi et al. (2017) demonstrated a related learning rule using integrate and fire neurons. The application focus of our work is different from that of Samadi et al. (2017), and the learning algorithm has important differences: Samadi et al. (2017) uses trigonometric functions and updates at every timestep. In contrast, our work demonstrates learning in an end-to-end spike-driven fashion and realizable using only additions and comparisons. Also, we demonstrated a stochastic version of eRBP, which lead to significantly better accuracies on MNIST (2.02% vs. 2.95% for 1,000 hidden neurons total).

### 3.3. Relation to prior work in spiking deep neural networks

Several approaches successfully realized the mapping of pre-trained artificial neural networks onto spiking neural networks using a firing rate code (O'Connor et al., 2013; Cao et al., 2014; Neftci et al., 2014; Das et al., 2015; Diehl et al., 2015; Hunsberger and Eliasmith, 2015; Marti et al., 2015; Esser et al., 2016; O'Connor and Welling, 2016) Such mapping techniques have the advantage that they can leverage the capabilities of existing machine learning frameworks such as Caffe (Jia et al., 2014) or Theano (Goodfellow et al., 2013) for brain-inspired computers. More recently, (Mostafa, 2016) used a temporal coding scheme where information is encoded in spike times instead of spike rates and the dynamics are cast in a differentiable form. As a result, the network can be trained using standard gradient descent to achieve very accurate, sparse and power-efficient classification. Although eRBP achieves comparable results, their approach naturally leads to sparse activity in the hidden layer which can be more advantageous in large and deep networks.

An intermediate approach is to learn online with standard BP using spike-based quantization of network states (O'Connor and Welling, 2016) and the instantaneous firing rate of the neurons (Lee et al., 2016). O'Connor and Welling (2016) eschews neural dynamics and instead operates directly on event-based (spiking) quantizations of vectors. Using this representation, common neural network operations including online gradient BP are mapped on to basic addition, comparison, and indexing operations applied to streams of signed spikes. As in eRBP, their learning rule achieves better results when weight updates are made in an event-based fashion, as this allows the network to update its parameters many times during the processing of a single data sample. Lee et al. (2016) propose a method for training spiking neural networks via a formulation of the instantaneous firing rate of the neuron obtained by low-pass filtering the spikes. There, quantities that can be related to the postsynaptic potential (rather than mean rates) are used to compute the derivative of the activity of the neuron, which can provide a useful gradient for backpropagation. Esser et al. (2016) use multiple spiking convolutional networks trained offline to achieve near state-of-the-art classification in standard benchmark tasks. Their approach maps onto the all-digital spiking neural network architecture using trinary weights. For the above approaches, the eRBP learning rule presented here can be used as a drop-in replacement and can reduce the computational footprint of learning by simplifying the backpropagated chain path and by operating directly with locally available variables i.e., membrane potentials and spikes.

### 3.4. Relation to prior work in spike-driven plasticity rules

STDP has been shown to be very powerful in a number of different models and tasks related to machine learning (Thorpe et al., 2001; Nessler et al., 2013; Neftci et al., 2014). Although, the implementation of acausal updates (triggered by presynaptic firing) is typically straightforward in cases where presynaptic lookup tables are used, the implementation of causal updates (triggered by postsynaptic firing) can be challenging due to the requirement of storing a reverse look-up table. Several approximations of STDP exist to solve this problem (Galluppi et al., 2014; Pedroni et al., 2016), but require dedicated circuits.

Thus, there is considerable benefit in hardware implementations of synaptic plasticity rules that forego the causal updates. Such rules, which we referred to as spike-driven plasticity, can be consistent with STDP (Brader et al., 2007; Clopath et al., 2010; Qiao et al., 2015; Sheik et al., 2016a), especially when using dynamical variables that are representative of the pre- and postsynaptic firing rates (such as calcium or average membrane voltage).

A common feature among spike-driven learning rules is a modulation or gating with a variable that reflects the average firing rate of the neuron, for example through calcium concentration (Graupner and Brunel, 2012; Huayaney et al., 2016) or the membrane potential (Clopath et al., 2010; Sheik et al., 2016a) or both Brader et al. (2007). Sheik et al. (2016a) recently proposed a membrane-gated rule inspired by calcium and voltage-based rules with homeostasis for learning unsupervised spike pattern detection. Their rule statistically emulates pairwise STDP using presynaptic spike timing only and using additions and multiplications. Except for homeostasis, eRBP follows similar dynamics but potentiation and depression magnitudes are dynamic and determined by external modulation, and comparisons are made on total synaptic currents.

The two compartment neuron model used in this work is motivated by conductance-based dynamics in Urbanczik and Senn (2014) and previous neuromorphic realizations of two compartment mixed signal spiking neurons Park et al. (2014). Although, the spiking network used in this work is current-based rather than conductance-based, eRBP shares strong similarities to the three-factor learning rule employed in Urbanczik and Senn (2014). The latter is composed of three factors: an approximation of the prediction error, the derivative of the membrane potential with respect to the synaptic weight, and a positive weighting function that stabilizes learning in certain scenarios. The first factor corresponds to the error modulation, while the second and third factors roughly correspond to the presynaptic activity and the derivative of the activation function. The differences between eRBP and (Urbanczik and Senn, 2014) (besides from the *random* BP which was considered in Lillicrap et al., 2016) stems mainly from two facts: (1) the firing rate description used here for simplicity and for easier comparisons between artificial neural networks and spiking neural networks and (2) eRBP is fully event-based in the sense that weights are updated only when the presynaptic neurons spike.

## 4. Conclusions and future directions

This article demonstrates a local, event-based synaptic plasticity rule for deep, feed-forward neural networks achieving classification accuracies on par with those obtained using equivalent machine learning algorithms. The learning rule combines two features: (1) Algorithmic simplicity: one addition and two comparisons per synaptic update provided one auxiliary state per neuron and (2) Locality: all the information for the weight update is available at each neuron and the synapse. The combination of these two features enables synaptic plasticity dynamics for neuromorphic deep learning machines.

Our results lay out a key component for the building blocks of spike-based deep learning using neural and synaptic operations largely demonstrated in existing neuromorphic technology (Chicca et al., 2013; Park et al., 2014; Merolla et al., 2014). Together with the near SynOp-MAC parity observed in the learning experiments compared to GPUs (Figure 5), we can reasonably expect real-time deep learning machines that operate on at least 100x to 1,000x smaller energy budget compared to current GPU technologies.

One limitation eRBP is related to the “loop duration,” i.e., the duration necessary from the input onset to a stable response in the error neurons. This duration scales with the number of layers, raising the question whether eRBP can generalize for very deep networks without impractical delays. Future work currently in investigation is to augment eRBP using recently proposed synthetic gradients (Jaderberg et al., 2016; Czarnecki et al., 2017), whereby gradients are estimated before the output neurons respond. This technique has been successfully tested with feedback alignment and direct feedback alignment, and thus has high chances of success using eRBP.

It can be reasonably expected that the deep learning community will uncover many variants of random BP, including in recurrent neural networks for sequence learning and memory augmented neural networks. In tandem with these developments, we envision that such RBP techniques will enable the embedded learning of pattern recognition, attention, working memory, and action selection mechanisms which promise transformative hardware architectures for embedded computing.

This work has focused on unstructured, feed-forward neural networks and a single benchmark task across multiple implementations for ease of comparison. Limitations in deep learning algorithms are often invisible on “toy” datasets like MNIST (Liao et al., 2015). Existing literature suggests that that random BP could also work for unsupervised learning (e.g., using autoencoders, Lee et al., 2014) in deeper and convolutional networks, as well as more difficult datasets such as CIFAR10. RLandom BP was demonstrated to be effective in a variety of tasks and network structures (Liao et al., 2015; Baldi et al., 2016), including convolutional neural networks (Baldi et al., 2016). In principle, we do not see major roadblocks in applying eRBP to spike-based convolutional neural networks, provided that the neuromorphic architecture can support weight sharing at the level of the feature.

## 5. Methods

### 5.1. Derivation of event-driven random backpropagation

In artificial neural networks, the mean-squared cost function for one data sample in a single layer neural network is:

(3) $$\begin{array}{l} L=\frac{1}{2}\sum_{i}e_{i}^{2},\\ e_{i}=(y_{i}-l_{i}), \end{array}$$

where *e*_*i*_ is the error of prediction neuron *i*, $y_{i}=\phi \left(\underset{j}{\sum }w_{ij}x_{j}\right)$ is the activity of the prediction neuron *i* with activation function ϕ, **x** is the data sample and *l*_*i*_ is the label associated with the data sample. The task of learning is to minimize this cost over the entire dataset. The gradient descent rule in artificial neural networks is often used to this end by modifying the network parameters **w** in the direction opposite to the gradient:

(4) $$\begin{array}{l} w_{ij}[t+1]=w_{ij}[t]-\eta \frac{\partial }{\partial w_{ij}}L,\\ \text{where }\frac{\partial }{\partial w_{ij}}L=\phi^{\prime }\left(\sum_{j}w_{ij}x_{j}\right)e_{i}x_{j}, \end{array}$$

and where η is a small learning rate. In deep networks, i.e., networks containing one or more hidden layers, the weights of the hidden layer neurons are modified by backpropagating the errors from the prediction layer using the chain rule:

(5) $$\begin{array}{l} \frac{\partial }{\partial w_{ij}^{l}}L=\delta_{ij}^{l}y_{j}^{l-1},\\ \text{where }\delta_{ij}^{l}=\phi^{\prime }\left(\sum_{j}w_{ij}^{l}y_{j}^{l-1}\right)\sum_{k}\delta_{ik}^{l+1}w_{ik}^{l+1}, \end{array}$$

where the δ for the topmost layer is *e*_*i*_, as in Equation (4) and *y* at the bottommost layer is the data *x*. This update rule is the well-known gradient back propagation algorithm ubiquitously used in deep learning (Rumelhart et al., 1988). Learning is typically carried out in forward passes (evaluation of the neural network activities) and backward passes (evaluation of δs). The computation of δ requires knowledge of the forward weights, thus gradient BP relies on the immediate availability of a symmetric transpose of the network for computing the backpropagated errors $\delta_{ij}^{l}$. Often the access to this information funnels through a von Neumann bottleneck, which dictates the fundamental limits of the computing substrate.

In the random BP rule considered here, the BP term δ is replaced with:

(6) $$\delta_{RBP}^{l}=\phi^{\prime }\left(\sum_{j}w_{ij}^{l}y_{j}^{l-1}\right)\sum_{k}e_{k}g_{ik}^{l}$$

where $g_{ik}^{l}$ are fixed random numbers. This backpropagated term does not depend on layer *l*+1, and thus does not have the recursive structure as in standard BP (Equation 5) or feedback alignment (Lillicrap et al., 2016). This form was previously referred to as direct feedback alignment (Nø kland, 2016) or skipped RBP (Baldi et al., 2016) and was shown to perform equally well on a broad spectrum of tasks compared to non-skipped RBP. A detailed justification of random BP is out of the scope of this article, and interested readers are referred to (Baldi et al., 2016; Lillicrap et al., 2016; Nø kland, 2016).

In the context of models of biological spiking neurons, RBP is appealing because it circumvents the problem of calculating the backpropagated errors and does not require bidirectional synapses or symmetric weights. RBP works remarkably well in a wide variety of classification and regression problems, using supervised and unsupervised learning in feed-forward networks, with a small penalty in accuracy.

The above BP rules are commonly used in artificial neural networks, where neuron outputs are represented as single scalar variables. To derive an equivalent spike-based rule, we start by matching this scalar value is the neuron's instantaneous firing rate. The cost function and its derivative for one data sample is then:

(7) $$\begin{array}{l} \;L_{sp}=\frac{1}{2}\sum_{i}{\left(\nu_{i}^{P}(t)-\nu_{i}^{L}(t)\right)}^{2}\\ \frac{\partial }{\partial w_{ij}}L_{sp}=\sum_{i}e_{i}(t)\frac{\partial }{\partial w_{ij}}\nu_{i}^{P}(t) \end{array}$$

where *e*_*i*_(*t*) is the error of prediction unit *i* and ν^*P*^, ν^*L*^ are the firing rates of prediction and label neurons, respectively.

Random BP (Equation 6) is straightforward to implement in artificial neural network simulations. However, spiking neurons and synapses, especially with the dynamics that can be afforded in low-power neuromorphic implementations typically do not have arbitrary mathematical operations at their disposal. For example, evaluating the derivative ϕ can be difficult depending on the form of ϕ and multiplications between the multiple factors involved in RBP can become very costly given that they must be performed at every synapse for every presynaptic event.

In the following, we derive an event-driven version of RBP that uses only two comparisons and one addition for each presynaptic spike to perform the weight update. The derivation proceeds as follows: (1) Derive the firing rate ν, i.e, the equivalent of ϕ in the spiking neural network, (2) Compute its derivative $\frac{\partial }{\partial w_{ij}}\nu_{i}\left(t\right)$, (3) Introduce modulation with a random linear combination of the classification error to the hidden neurons, (4) Devise a plasticity rule that increments the weight with the product of the latter two factors times the presynaptic activity.

#### Activation function of spiking neurons with background poisson noise and its derivative

The dynamics of spiking neural circuits driven by Poisson spike trains is often studied in the diffusion approximation (Wang, 1999; Brunel and Hakim, 1999; Fusi and Mattia, 1999; Brunel, 2000; Renart et al., 2003; Tuckwell, 2005; Deco et al., 2008). In this approximation, the firing rates of individual neurons are replaced by a common time-dependent population activity variable with the same mean and two-point correlation function as the original variables, corresponding here to a Gaussian process. The approximation is true when the following assumptions are verified: (1) the charge delivered by each spike to the postsynaptic neuron is small compared to the charge necessary to generate an action potential, (2) the number of inputs to each neuron is large, (3) the spike times are uncorrelated. In the diffusion approximation, only the first two moments of the synaptic current are retained. The currents to the neuron, *I*(*t*), can then be decomposed as:

(8) I(t)=μ+σω(t),

where $\mu =\langle I\left(t\right)\rangle =\underset{j}{\sum }w_{j}\nu_{j}$ and $\sigma^{2}=w_{bg}^{2}\nu_{bg}$, where ν_*bg*_ is the firing rate of the background activity, and ω(*t*) is the white noise process. We restrict neuron dynamics to the case of synaptic time constants that are much larger than the membrane time constant, i.e., τ_*m*_ ≪ τ_*syn*_, such that we can neglect the fluctuations caused by synaptic activity from other neurons in the network i.e., σ is constant. Although, the above dynamics are not true in general, in a neuromorphic approach, the parameters can be chosen accordingly during configuration or at design.

In this case, the neuron's membrane potential dynamics is an Ornstein-Uhlenbeck (OU) process (Gardiner, 2012). The stationary distribution of the freely evolving membrane potential (no firing threshold) is a Gaussian distribution:

(9) $$V_{nt}\sim N(\frac{\mu }{g_{L}},\frac{\sigma^{2}}{2g_{L}^{2}\tau_{m}}).$$

where *g*_*L*_ is the leak conductance and τ_*m*_ is the membrane time constant. Although, this distribution is generally not representative of the membrane potential of the I&F neuron due to the firing threshold (Gerstner and Kistler, 2002), the considered case τ_*m*_ ≪ τ_*syn*_ yields approximately a truncated Gaussian distribution, where neurons with *V*_*nt*_ > 0 fire at their maximum rate of $\frac{1}{\tau_{refr}}$. This approximation is less exact for very large μ due to the resetting, but the resulting form highlights the essence of eRBP while maintaining mathematical tractability. Furthermore, using a first-passage time approach, Petrovici et al. (2013) computed corrections that account for small synaptic time constants and the effect of the firing threshold on this distribution.

The firing rate of neuron *i* is approximately equal to the inverse of the refractory period, $\nu_{i}=\tau_{refr}^{-1}$ with probability *P*(*V*_*nt, i*_(*t* + 1) ≥ 0|**s**(*t*)) and zero otherwise. The probability is equal to one minus the cumulative distribution function of *V*_*nt, i*_:

$$P(V_{nt,i}(t+1)\geq 0|s(t))=\frac{1}{2}\left(1+\mathrm{erf}\left(\frac{\mu_{i}(t)}{\sigma_{OU}\sqrt{2}}\right)\right),$$

where “erf” stands for the error function. The firing rate of neuron *i* becomes:

(10) $$\nu_{i}=\frac{1}{\tau_{refr}}\frac{1}{2}\left(1+\mathrm{erf}\left(\frac{\sqrt{\tau_{m}}}{\sigma }\sum_{j}w_{ij}\nu_{j}\right)\right).$$

For gradient descent, we require the derivative of the neuron's activation function with respect to the weight *w*. By definition of the cumulative distribution, this is the Gaussian function in Equation (9) times the presynaptic activity:

(11) $$\frac{\partial }{\partial w_{ij}}\nu_{i}\;\propto \frac{1}{\sigma_{OU}\sqrt{2\pi }}\exp\left(-\frac{\mu_{i}{\left(t\right)}^{2}}{2\sigma_{OU}^{2}}\right)\nu_{j}(t).$$

As in previous work (Neftci et al., 2014), we replace ν_*j*_(*t*) in the above equations with the presynaptic spike train *s*_*j*_(*t*) (modeled as a sum of delta Dirac functions) to obtain an asynchronous, *event-driven* update, where the derivative is evaluated only when the presynaptic neuron spikes. This approach is justified by the fact that the learning rate is typically small, such that the event-driven updates are averaged at the synaptic weight variable (Gerstner and Kistler, 2002). Thus the derivative becomes:

(12) $$\frac{\partial }{\partial w_{ij}}\nu_{i}\propto \{\begin{array}{l} \exp(-\frac{\mu_{i}{\left(t\right)}^{2}}{2\sigma_{OU}^{2}})\text{ if pre-synaptic neuron }j\text{ spiked}\\ 0\text{ otherwise} \end{array}.$$

In the considered spiking neuron dynamics, the Gaussian function is not directly available. Although, a sampling scheme based on the membrane potential to approximate the derivative is possible, here we follow a simpler solution: Backed by extensive simulations, and inspired by previously proposed learning rules based on membrane potential gated learning rules (Brader et al., 2007; Clopath et al., 2010; Sheik et al., 2016a), we find that replacing the Gaussian function with a boxcar function Θ operating on the total synaptic input, *I*(*t*), with boundaries *b*_*min*_ and *b*_*max*_ yields results that are as good as using the exact derivative. With appropriate boundaries, Θ(*I*(*t*)) can be interpreted as a piecewise constant approximation of the Gaussian function^2^ since *I*(*t*) is proportional to its argument $\underset{j}{\sum }w_{ij}\nu_{j}$, and has the advantage that an explicit multiplication with the modulation is unnecessary in the random BP rule (explained below).

(13) $$\frac{\partial }{\partial w_{ij}}\nu_{i}\propto \{\begin{array}{l} 1\text{ if pre-synaptic neuron j spiked and }b_{min}\;<\;I_{i}(t)\\ <b_{max}\\ 0\text{ otherwise} \end{array}$$

The resulting derivative function is similar in spirit to straight-through estimators used in machine learning (Courbariaux and Bengio, 2016).

#### Derivation of event-driven random backpropagation

For simplicity, the error *e*_*i*_(*t*) is computed using a pair of spiking neurons with a rectified linear activation function. One neuron computes the positive values of *e*_*i*_(*t*), while the other neuron computes the negative values of *e*_*i*_(*t*) such that:

(14) $$\begin{array}{l} \nu_{i}^{E+}(t)\propto \nu_{i}^{P}(t)-\nu_{i}^{L}(t),\\ \nu_{i}^{E-}(t)\;\propto -\nu_{i}^{P}(t)+\nu_{i}^{L}(t). \end{array}$$

Each pair of error neurons synapse with a leaky dendritic compartment *U* of the hidden and prediction neurons using equal synaptic weights with opposite sign, generating a dendritic potential proportional to $\left(\nu_{i}^{E+}\left(t\right)-\nu_{i}^{E-}\left(t\right)\right)≅e_{i}$. Several other schemes for communicating the errors are possible. For example an earlier version of eRBP used a positively biased error neuron per class (rather than a positive negative pair as above) such that the neuron operated (mostly) in the linear regime. This solution led to similar results but was computationally more expensive due to error neurons being strongly active even when the classification was correct. Population codes of heterogeneous neurons as in Salinas and Abbott (1994) and Eliasmith and Anderson (2004) may provide even more flexible dynamics for conveying errors. The weight update for the last layer becomes:

(15) $$\Delta w_{ij}\propto \{\begin{array}{l} -e_{i}(t)\text{ if pre-synaptic neuron }j\text{ of layer }h\;\\ \text{ spiked and }b_{min}<I_{i<b_{max}}\\ 0\text{ otherwise} \end{array}.$$

The weight update for the hidden layers is similar, except that a random linear combination of the error is used instead of *e*_*i*_:

(16) $$\Delta w_{ij}^{C}\text{​}\propto \text{​​}\{\begin{array}{l} -\sum_{k}g_{ik}e_{k}^{E}(t)\text{ if pre-synaptic neuron }j\text{ of}\\ \text{layer }d,\;h\text{ spiked and }b_{min}<I_{i}<b_{max}\\ 0\text{ otherwise} \end{array}.$$

All weight initializations are scaled with the number of rows and the number of columns as $g_{ik}\sim U\left(\sqrt{\frac{6}{N_{E}+N_{H}}}\right)$ (Glorot and Bengio, 2010), where *N*_*E*_ is the number of error neurons and *N*_*H*_ is the number of hidden neurons.

In the following, we detail the spiking neuron dynamics that can efficiently implement eRBP.

### 5.2. Spiking neural network and plasticity dynamics

The network used for eRBP consists of one or two feed-forward layers (Figure 1) with *N*_*d*_ “data” neurons, *N*_*h*_ hidden neurons and *N*_*p*_ prediction neurons. The top layer, labeled *P*, is the prediction. The feedback from the error population is fed back directly to the hidden layers' neurons. The network is composed of three types of neurons:

(1) **Error-coding neurons** are non-leaky integrate and fire neurons following the linear dynamics:

(17) $$\begin{array}{l} \;C\frac{\text{d}}{\text{d}t}V_{i}^{E+}=w^{L+}(s_{i}^{P}(t)-s_{i}^{L}(t))\\ \text{if }V^{E+}>V_{T}^{E}\text{ then }V^{E+}\leftarrow V^{E+}-V_{T}^{E}, \end{array}$$

where $s_{i}^{P}\left(t\right)$ and $s_{i}^{L}\left(t\right)$ are spike trains from prediction neurons and labels (teaching signal). In addition, the membrane potential is lower bounded to $V_{T}^{E}$ to prevent negative activity to accumulate across trials. Each error neuron has one counterpart neuron with weights of opposite sign, i.e., *w*^*L*−^ = −*w*^*L*+^ to encode the negative errors. The firing rate of the error-coding neurons is proportional to a linear rectification of the inputs. For simplicity, the label spike train is regular with firing rate equal to $\tau_{refr}^{-1}$. When the prediction neurons classify correctly, $\left(s_{i}^{P}\left(t\right)-s_{i}^{L}\left(t\right)\right)≅0$, such that the error neurons remain silent.

(2) **Hidden neurons** follow current-based I&F dynamics:

(18) $$\begin{array}{l} C\frac{\text{d}}{\text{d}t}\left(\begin{array}{c} V_{i}^{h}\\ U_{i}^{h} \end{array}\right)=-\left(\begin{array}{c} g_{V}V_{i}^{h}\\ g_{U}U_{i}^{h} \end{array}\right)+\left(\begin{array}{c} I_{i}^{h}+\sigma_{w}\omega_{i}^{h}(t)\\ \sum_{k=1}^{N_{L}}g_{ik}^{E+}s_{k}^{E+}(t)-g_{ik}^{E-}s_{k}^{E-}(t) \end{array}\right)\\ \tau_{syn}\frac{\text{d}}{\text{d}t}I_{i}^{h}=-I_{i}^{h}+\sum_{k=1}^{N_{d}}w_{ik}^{d}s_{k}^{d}(t)\xi (t)+\sum_{j=1}^{N_{h}}w_{ij}^{h}s_{j}^{h}(t)\xi (t)\\ \text{if }V_{i}^{h}(t)>V_{T}\text{ then }V_{i}^{h}\leftarrow 0\text{ during refractory period }\tau_{refr}. \end{array}$$

where $s_{k}^{d}\left(t\right)$ and $s_{j}^{h}\left(t\right)$ are the spike trains of the data neurons and the hidden neurons, respectively, *I*^*h*^ are current-based synapse dynamics, $\sigma_{w}\omega_{i}^{h}\left(t\right)$ a Poisson process of rate 1 kHz and amplitude σ_*w*_, and ξ is a stochastic Bernouilli process with probability (1−*p*) (indices *i, j* are omitted for clarity). The Poisson process simulates background Poisson activity and contributes additively to the membrane potential, whereas the Bernouilli process contributes multiplicatively by randomly “blanking-out” the proportion *p* of the input spikes. In this work, we consider feed-forward networks, i.e., the weight matrix *w*^*h*^ is restricted to be upper diagonal. Each neuron is equipped with a separate “dendritic” compartment $U_{i}^{h}$ following similar subthreshold dynamics as the membrane potential and where *s*^*E*^(*t*) is the spike train of the error-coding neurons and $g_{ij}^{E}$ is a fixed random matrix. The dendritic compartment is not directly coupled to the “somatic” membrane potential $V_{i}^{h}$, but indirectly through the learning dynamics. For every hidden neuron *i*, $\underset{j}{\sum }w_{ij}^{E}=0$, ensuring that the spontaneous firing rate of the error-coding neurons does not bias the learning. The synaptic weight dynamics follow a dendrite-modulated and gated rule:

(19) $$\frac{\text{d}}{\text{d}t}w_{ij}^{h}=\eta U_{i}^{h}\Theta (I_{i}^{h})s_{j}^{h}(t).$$

where Θ is a boxcar function with boundaries *b*_*min*_ and *b*_*max*_ and η is the learning rate.

(3) **Prediction neurons**, synapses and synaptic weight updates follow the same dynamics as the hidden neurons except for the dendritic compartment, and one-to-one connection with pairs of error-neurons associated to the same class:

(20) $$C\frac{\text{d}}{\text{d}t}\;\left(\begin{array}{c} V_{i}^{P}\\ U_{i}^{P} \end{array}\right)=-\;\left(\begin{array}{c} g_{V}V_{i}^{P}\\ g_{U}U_{i}^{P} \end{array}\right)+\left(\begin{array}{c} I_{i}^{P}+\sigma_{w}\omega_{i}^{P}(t)\\ w^{E}s_{i}^{E+}(t)-w^{E}s_{i}^{E-}(t) \end{array}\right).$$

**Figure 7:**
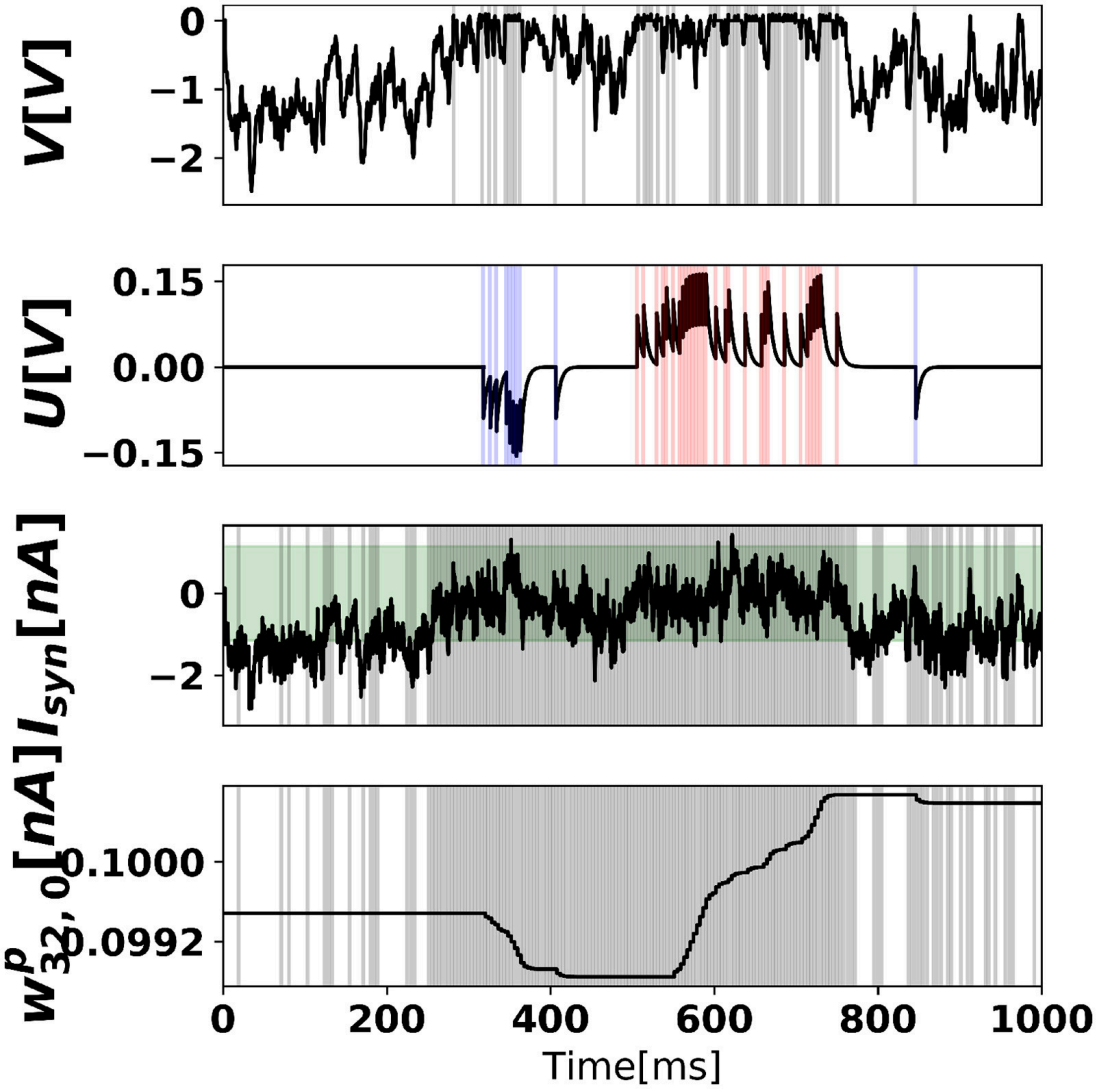
Neural states and synaptic weight of the prediction neuron after 500 training examples. **(Top)** Somatic membrane potential dynamics of prediction neuron 0, where output spikes are superimposed as gray vertical bars. **(Middle-top)** Dendritic membrane potential, where blue and red bars indicate negative error neuron 0 spikes and positive error neuron 0 spikes, respectively. In the time range (500,750), the digit 0 is presented to the network. **(Middle-bottom)** Total synaptic current of prediction neuron 0, where superimposed vertical bars are presynaptic spikes of hidden neuron 32. The green shaded area (*b*_*min*_, *b*_*max*_) corresponds to the plasticity-enabled region, i.e., the approximate derivative function Θ(*I*^*p*^(*t*)). **(Bottom)** Synaptic weight between hidden neuron 32 and prediction neuron 0.

The spike trains at the data layer were generated using a stochastic neuron with instantaneous firing rate [exponential hazard function (Gerstner and Kistler, 2002) with absolute refractory period]:

(21) $$\nu^{d}(d,t-t^{\prime })=\{\begin{array}{ll} 0 & if\;t-t^{\prime }<\tau_{refr}\\ \tau_{refr}^{-1}\exp(\beta d+\gamma ) & t-t^{\prime }\geq \tau_{refr} \end{array},$$

where *d* is the intensity of the pixel (scaled from 0 to 1), and *t*′ is the time of the last spike. Figure 7 illustrates the neural dynamics in a prediction neuron, in a network trained with 500 training samples (1/100 of an epoch).

#### 5.2.1. Stochastic, blank-out synapses

In practice, we find that neurons tend to strongly synchronize in late stages of the training. The analysis provided above does not accurately describe synchronized dynamics, since one of the assumptions for the diffusion approximation is that spike times are uncorrelated. Multiplicative stochasticity was previously shown to be beneficial for regularization and decorrelation of spike trains, while being easy to implement in neuromorphic hardware (Neftci E. et al., 2016). Following the ideas of synaptic sampling (Neftci E. et al., 2016), we find that replacing the background Poisson noise with multiplicative, blank-out noise (Vogelstein et al., 2002) at the plastic synapses slightly improves the results and mitigates the energetic footprint of the stochasticity (Sheik et al., 2016b).

### 5.3. Experimental setup and software simulations

We trained fully connected feed-forward networks on two datasets, the standard MNIST hand-written digits (LeCun et al., 1998) and EMNIST hand-written digits and letters (Cohen et al., 2017). The EMNIST dataset is a variant of the full NIST database and consists of digits, uppercase and lowercase handwritten letters. We used the balanced dataset which contains a balanced subset of 47 classes (10 digits and 37 letters), where classes were chosen to avoid classification errors resulting purely from misclassification between uppercase and lower-case letters. Namely, classes for letters c, i–m, o, p, s, u–z were merged with their uppercase counterparts. The MNIST dataset was separated in three groups, training, validation, and testing (50,000, 10,000, 10,000 samples, respectively) and the EMNIST dataset was separated in two groups, training and testing (112,800, 18,800 for EMNIST). We did not use a validation set for EMNIST as we applied the same hyperparameters obtained from MNIST (except for *N*_*l*_). Spiking simulations were run for five different seeds (in one hidden layer networks) and only one seed for two-layer networks. Test error results were obtained averaging test errors across the last 5 epochs (for MNIST and EMNIST). For computational reasons, the image pixel intensities in MNIST and EMNIST were converted into spikes by driving an SRM neuron with parameters matched to those of the network I&F neurons. The intensities driving the SRM were scaled and shifted. Label neuron intensities were scaled such that the associated SRM neurons responded regularly, and image neuron intensities were scaled and shifted in such a way that neurons with zero intensity did not spike on visual inspection. Except for label neurons and provided that the first layer neurons were sufficiently active, we did not observe any substantial effects of the scaling on the learning.

To keep the durations of the spiking simulations tractable, learning was run for 60 epochs (MNIST) or 30 epochs (EMNIST), compared to 1,000 epochs in the GPU. This is not a major limitation since errors appear to converge earlier in the spiking neural network. During a training epoch, each of the training digits were presented in during 250*ms*. We tested eRBP using two configurations: one with additive noise (σ_*w*_ > 0, *p* = 0, labeled eRBP_+_), and one with multiplicative noise implemented as blank-out noise on the connections (blank-out probability *p* = 0.45 and σ_*w*_ = 0, labeled eRBP_×_). For the spiking implementation, the reported results were obtained using (hyper)parameters obtained from a coarse grid search and a manual search. The parameter search swept the learning rate η, blank-out probability *p*, the learning gating values *b*_*min*_ and *b*_*max*_, and the magnitude of the random and initialization weights. Parameters for BP were identical to those used in Lee et al. (2014) and the learning rate of the (GPU-based) RBP simulations was manually adjusted to achieve peak accuracy on the 784-200-10 network.

All learning rates were kept fixed during the simulation. Other *I&F* neuron related parameters were carried over from previous work (Neftci E. O. et al., 2016) and not specifically tuned for eRBP. To prevent the network from learning (spurious) transitions between digits, the synaptic weights did not update in the first 50*ms* window of each digit presentation.

We tested eRBP training on a spiking neural network based on the Auryn simulator (Zenke and Gerstner, 2014). Results are compared against GPU implementations of RBP and standard BP performed in Theano (Bergstra et al., 2010) using an equivalent, non-spiking neural network. Parameters used in the Auryn and Theano simulations are provided in Table 2. Besides layered connectivity, all networks were unstructured i.e., no convolutions or poolings).

**Table 2:** Parameters used for the continuous-time spiking neural network simulation implementing eRBP.

*N*_*d*_	Number of data neurons	All networks	784
*N*_*h*_	Number of hidden neurons	All networks	100,200,400,1000
*N*_*l*_	Number of label neurons	All networks	10
*N*_*E*+_	Number of positive error neurons	All networks	10
*N*_*E*−_	Number of negative error neurons	All networks	10
*N*_*p*_	Number of prediction neurons	All networks	10
σ	Poisson noise weight	eRBP_+_	50· 10^−3^ *nA*
		eRBP_×_	0· 10^−3^ *nA*
*p*	Blank-out probability	eRBP_+_	1.0
		eRBP_×_	0.45
τ_*refr*_	Refractory period	Prediction and hidden neurons	3.9 *ms*
		Data neurons	4.0 *ms*
τ_*syn*_	Synaptic Time Constant	All synapses	4 *ms*
*g*_*V*_	Leak conductance state *V*	Prediction and hidden neurons	1 *nS*
*g*_*U*_	Leak conductance state *U*	Prediction and hidden neurons	5 *nS*
*C*	Membrane capacitance	All neurons	1 *pF*
*V*_*T*_	Firing threshold	Prediction and Hidden neurons	100 *mV*
$V_{T}^{E}$		Error neurons	100 *mV*
*N*_*train*_	Number of training samples used	All figures	50000
*N*_*test*_	Number of training samples used	Table 1 eRBP_+_, eRBP_×_	10000
		Table 2 eRBP_+_, eRBP_×_	1000
		Table 2 RBP, BP	10000
*T*_*train*_	Training sample duration	All models	100 *mV*
*T*_*test*_	Testing sample duration	Table 1, Figure 4	500 *ms*
		Table 2	250 *ms*
*w*^*h*^, *w*^*d*^, *w*^*p*^, *g*	Initial weight matrix	RBP, BP	$U(\sqrt{\frac{6}{\#rows+\#cols}})\text{nA}$
		eRBP_+_	$U(\sqrt{\frac{6}{\#rows+\#cols}})\text{nA}$
		eRBP_×_	$U(\sqrt{\frac{7}{\#rows+\#cols}})\text{nA}$
*w*^*E*^		eRBP_+_, eRBP_×_	90· 10^−3^nA
*w*^*L*+^		eRBP_+_, eRBP_×_	90· 10^−3^nA
*w*^*L*−^		eRBP_+_, eRBP_×_	−90· 10^−3^nA
*b*_*min*_,*b*_*max*_		eRBP_+_, eRBP_×_	−1.15, 1.15 *nA*
	2nd hidden layer	eRBP_+_, eRBP_×_	-25, 25 *nA*
	Figure 6	eRBP_+_, eRBP_×_	−0.6, 0.6 *nA*
β	Data neuron input scale	eRBP_+_, eRBP_×_	0.5
γ	Data neuron input threshold	eRBP_+_, eRBP_×_	−0.215
η	Learning Rate	eRBP_+_	6· 10^−4^nS
		eRBP_×_	10· 10^−4^nS
		RBP, BP	0.4/*n*_*batch*_
*n*_*batch*_	Minibatch size	RBP(100), BP(100)	100
		RBP(1), BP(1)	1

## Author contributions

EN and GD: Designed experiment, conducted experiments, and wrote the paper. EN, GD, SP, and CA: Contributed software and tools.

### Conflict of interest statement

The authors declare that the research was conducted in the absence of any commercial or financial relationships that could be construed as a potential conflict of interest.

## Footnotes

^1^Such logical “and” operation on top of a graded signal was previously used for conditional signal propagation in neuromorphic VLSI spiking neural networks (Neftci et al., 2013).

^2^or equivalently, for the purpose of the derivative evaluation, the activation function is approximated as a rectified linear with hard saturation at $\tau_{refr}^{-1}$, also called “hard tanh” in the machine learning community.
